# After the Shock: Impact of Ectopic Pregnancy on Subsequent Fertility and Parenthood

**DOI:** 10.3390/biomedicines13092205

**Published:** 2025-09-09

**Authors:** Efthalia Moustakli, Ekaterini Domali, Anastasios Potiris, Angeliki Gerede, Ismini Anagnostaki, Athanasios Zikopoulos, Charalampos Theofanakis, Nikolaos Kathopoulis, Konstantinos Louis, Peter Drakakis, Sofoklis Stavros

**Affiliations:** 1 Department of Nursing, School of Health Sciences, University of Ioannina, 4th kilometer National Highway Str. Ioannina-Athens, 45500 Ioannina, Greece; thaleia.moustakli@gmail.com; 2 First Department of Obstetrics and Gynecology, Alexandra Hospital, Medical School, National and Kapodistrian University of Athens, 11528 Athens, Greece; kdomali@yahoo.fr (E.D.); nickatho@gmail.com (N.K.); 3 Third Department of Obstetrics and Gynecology, Medical School, University General Hospital “ATTIKON”, National and Kapodistrian University of Athens, 12462 Athens, Greece; apotiris@med.uoa.gr (A.P.); isanagnostaki3@gmail.com (I.A.); thanzik92@gmail.com (A.Z.); kostaslouisss@gmail.com (K.L.); pdrakakis@med.uoa.gr (P.D.); 4 Department of Obstetrics and Gynecology, Democritus University of Thrace, Alexandroupolis Campus, 69100 Alexandroupolis, Greece; agerede@otenet.gr

**Keywords:** ectopic pregnancy, fertility, parenthood, early pregnancy, holistic approach

## Abstract

The potentially fatal condition known as ectopic pregnancy (EP) occurs when an embryo implants outside of the uterus, usually in the fallopian tube. It accounts for approximately 1–2% of all pregnancies and remains a leading cause of maternal morbidity in the first trimester. EP is an important area of focus in reproductive health that extends beyond its immediate clinical care. The purpose of this study is to investigate the effects of EP on the physical, reproductive, and psychological aspects of eventual fertility and parental outcomes. The findings from qualitative interviews, case–control studies, and cohort studies that have been published in peer-reviewed journals over the past 20 years were compiled into a narrative literature review. Included were studies looking at patient experiences after EP, psychosocial impacts, and reproductive results. According to research, women who have had EP in the past may have a slightly lower chance of becoming pregnant in the future, particularly following a salpingectomy. Assisted reproductive technology may potentially mitigate some of these risks. The parenting journey is often complicated by psychological consequences. Access to fertility services and counseling was found to have a significant impact on post-EP reproductive outcomes. The need for thorough follow-up care that addresses both physical and mental wellness is highlighted by the fact that EP can have long-lasting impacts on fertility and the parenting path. To optimize patient well-being and reproductive results, post-EP treatment must include early fertility counseling and psychological support.

## 1. Introduction

Approximately 1% to 2% of all reported pregnancies are affected by EP, which continues to be a major global reproductive health concern. EP can be lethal if it is not detected and treated quickly [1]. Fertilized ovum implantation outside the uterus, typically in the fallopian tube, is known as EP. Despite improvements in early detection and treatment outcomes due to advances in diagnostic imaging and medical care, the EP experience often leaves affected individuals and couples with long-lasting physical and emotional effects [2].

Importantly, the burden of EP is not evenly distributed worldwide. Widespread availability of serum β-hCG tests and ultrasonography allows for earlier diagnosis and reduced maternal mortality in high-resource environments [3]. Women in areas with minimal resources, however, experience increased case fatality rates, delayed diagnosis, and restricted access to emergency care. These regional differences draw attention to the injustices in reproductive health that exist around the world and emphasize the necessity of context-sensitive methods for managing and monitoring EP [4].

Future fertility is significantly impacted by EP, in addition to the immediate risks of bleeding and tubule damage [1]. A woman’s fertility may be directly impacted by the loss of a pregnancy, which is frequently unanticipated and unpleasant, as well as potential surgical procedures (like a salpingectomy) that compromise tubal function or result in unilateral tubal loss. Even when medical or conservative treatment is effective, the risk of recurrent EP and long-term infertility still exists for many women [5]. These risks are further influenced by socioeconomic status, ethnicity, and access to healthcare: women who have less access to follow-up monitoring or assisted reproductive technologies (ART) are frequently more likely to encounter obstacles in their future motherhood [6].

Furthermore, the psychological effects of EP are frequently disregarded in professional care [7]. Emotional responses like grief, anxiety, sadness, and fear of recurrence can influence decisions about future attempts at conception, delay subsequent pregnancies, or even result in a complete avoidance of motherhood. Couples may undergo relationship distress and change their reproductive objectives as a result of this experience [8,9]. Integrated psychological support is essential, as demonstrated by these clinically important results.

EP frequently carries deeper social and cultural implications that extend beyond the person. Infertility and pregnancy loss can be stigmatized in some situations, which can cause marital stress, partner blame, or social rejection [10]. The way women and couples grieve EP and later make reproductive choices is also shaped by cultural differences in how grief is expressed and coping mechanisms. To properly represent the varied realities of reproductive health around the world and to avoid presenting all women as a homogenous group, it is imperative to take different viewpoints into account [11].

The long-term effects of EP on reproduction and the ensuing psychological effects remain largely unknown, despite a greater understanding of these challenges [12]. Acute care and recurrence rates are the main topics of the majority of the currently published material, with little consideration given to patient-centered outcomes such as the time to subsequent conception, the desire to become a parent in the future, and the quality of life after the incident [13,14]. These results can be framed by theoretical frameworks from the fields of sociology, health psychology, and reproductive justice, which highlight how identity, coping strategies, and institutional injustices interact with biological healing [15].

This narrative review aims to provide a comprehensive examination of the effects of EP on subsequent fertility and the journey to motherhood. By integrating patient experiences with existing evidence, we aim to inform medical practice, enhance counseling approaches, and highlight gaps requiring further research to support affected individuals in reproductive decision-making and psychological recovery.

## 2. Materials and Methods

### 2.1. Literature Search Strategy

The purpose of this narrative review was to compile the most recent data regarding EP’s effects on later fertility and the desire to become a parent. To provide a comprehensive overview of the medical, reproductive, and psychological effects that accompany an EP, a large body of published research was examined.

A non-systematic search of the literature was conducted using electronic databases including Google Scholar, PubMed, Embase, and Scopus. Structured, peer-reviewed coverage was offered by PubMed, Embase, and Scopus; however, Google Scholar was not a primary source and was primarily utilized for supplemental “grey literature” (such as guidelines and conference papers).

The search covered publications from January 2000 to June 2025. The year 2000 was chosen as a cutoff to capture studies conducted after the widespread introduction of high-resolution ultrasonography, β-hCG monitoring, and laparoscopic surgery, which significantly altered diagnosis and management pathways. More recent studies published after 2015 were given particular consideration to ensure current relevance.

Search strings combined Medical Subject Headings (MeSH) and free-text terms. Examples included combinations such as (“ectopic pregnancy” OR “tubal pregnancy”) AND (“fertility” OR “infertility” OR “reproductive outcomes”), (“ectopic pregnancy” OR “pregnancy loss”) AND (“psychological impact” OR “emotional well-being” OR “PTSD” OR “depression” OR “anxiety”), and (“ectopic pregnancy”) AND (“assisted reproductive technology” OR “IVF” OR “ART” OR “parenthood” OR “decision-making”). Boolean operators (“AND,” “OR”), truncation, and filters for year, human studies, and English language were applied. Manual searches of reference lists from relevant articles, review papers, and clinical practice guidelines (the “snowball method”) were also performed.

**Figure 1:**
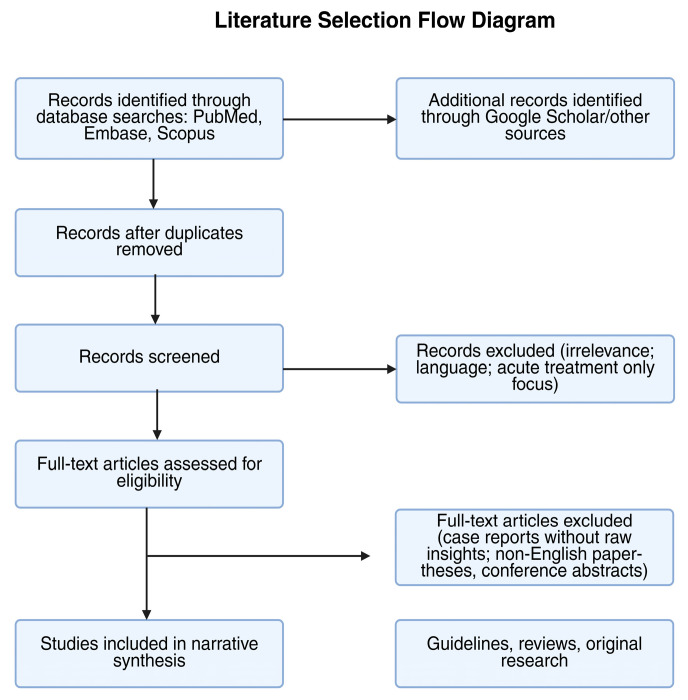
Flow diagram illustrating the identification, screening, and inclusion of studies. Records were obtained from PubMed, Embase, Scopus, and supplementary grey literature from Google Scholar. After exclusions, eligible full texts were synthesized narratively by thematic domain.

Relevant data from the selected literature were extracted and synthesized narratively. The findings were organized thematically according to key areas of focus, including pathophysiology and risk factors, impact on fertility, emotional and psychological consequences, and the decision-making process related to parenthood following an EP. Where available, statistical data such as live birth rates and recurrence rates were incorporated to support the discussion (Figure 1).

### 2.2. Inclusion and Exclusion Criteria

Clinical practice guidelines, systematic reviews, meta-analyses, and original research publications (qualitative, quantitative, and randomized controlled trials) were among the acceptable sources. Unless they provided new clinical or psychological insights, case reports were not included. Research was also disqualified if it solely addressed the acute treatment of EP without taking psychosocial or reproductive consequences into account. Theses, conference abstracts, and unpublished research were not taken into account unless they were listed in peer-reviewed databases. We recognize that the inclusion of only English-language research may introduce linguistic bias, especially in a condition of global importance; this limitation is discussed.

### 2.3. Data Extraction and Synthesis

Two authors retrieved pertinent information from the chosen literature and double-checked its accuracy. Reproductive outcomes (pregnancy rates, live birth rates, recurrence), psychological outcomes (PTSD, depression, anxiety, coping mechanisms), sociocultural factors (stigma, partner dynamics, access to care), study design, sample size, geographic setting, population characteristics, and treatment type were the main areas of extraction.

The results were compiled thematically. The identification of themes involved combining deductive categorization (based on pre-established domains: pathophysiology/risk factors, fertility impact, psychological implications, and parenting decision-making) with inductive refining, which involved incorporating new patterns found in the data. To prevent double-counting, context was provided by systematic reviews and meta-analyses, and unique data points were only offered by individual primary studies if they were not already combined in higher-level analyses.

### 2.4. Limitations

Instead of being a systematic review, this one was narrative in nature, which permits a more thorough integration of clinical, psychological, and sociocultural viewpoints but may compromise repeatability and transparency. Readers should consider this constraint when interpreting results because selection bias may be present. The inclusion of just English-language papers may have introduced linguistic bias by excluding pertinent research from non-English-speaking places where the burden of EP is significant. Furthermore, the relative quality of the included studies may differ because no systematic risk of bias assessment was carried out. The methodological rigor was qualitatively described and gaps in the literature were highlighted despite these constraints.

## 3. Pathophysiology and Risk Factors of EP

### 3.1. Etiology and Biological Mechanisms

EP occurs when a fertilized ovum implants outside the uterine cavity, most commonly within the fallopian tube, although implantation can also occur in other locations such as the cervix, ovary, abdominal cavity, or cesarean scar [16]. Several risk factors have been identified for EP, as summarized in Table 1. The underlying pathophysiological mechanism typically involves impaired tubal transport of the fertilized ovum. Coordinated ciliary action and smooth muscle contractions are necessary for normal tubal function in order to move the embryo toward the uterine cavity. A disruption in this process may cause the embryo to pass more slowly, which could result in an early implantation in the fallopian tube [17].

Modified tubal motility and dysfunction can be caused by a number of factors. Ciliary activity may be hindered by damage to the tubal epithelium after surgery or infection [29]. The risk of ectopic implantation is increased by inflammatory processes that can result in fibrosis, adhesions, and anatomical distortion. Hormonal abnormalities that impact endometrial receptivity or tubal motility may also be involved. Recent research has indicated that the pathophysiology of EP may also involve molecular and immunological factors, such as changed expression of adhesion molecules or cytokines within the fallopian tube [30].

### 3.2. Common Risk Factors

There are several known clinical risk factors that put women at risk for an EP. A history of pelvic inflammatory disease (PID), especially infections brought on by Chlamydia trachomatis or Neisseria gonorrhoeae, is one of the most important [31]. Loss of ciliary function and tubal scarring are possible outcomes of these infections. In a similar vein, previous tubal surgery, such as reconstructive procedures or tubal ligation, raises the risk by altering the typical tubal architecture [32].

A well-known risk factor is prior EP, with recurrence rates ranging from 10% to 25%, contingent on the degree of tubal damage and the type of treatment [33]. Women who become pregnant after using assisted reproductive technologies (ART), like in vitro fertilization (IVF), are also more vulnerable, perhaps as a result of the technicalities of embryo transfer or underlying tubal pathology [34].

It has been demonstrated that certain lifestyle choices, like smoking cigarettes, raise the risk of EP by changing tubal motility and compromising ciliary function. Although the precise causes are still unknown, EP has also been linked to advanced maternal age, especially in women over 35 [16]. Additionally, even while certain birth control methods, such as intrauterine devices (IUDs), are quite successful in preventing intrauterine pregnancy, they may nevertheless permit the uncommon occurrence of ectopic implantation if contraceptive failure occurs [35].

### 3.3. Diagnostic Challenges

Given its non-specific symptoms and variable presentation, EP is still difficult to diagnose in a timely manner. Amenorrhea, abdominal pain, and vaginal bleeding are typical clinical symptoms; however, these symptoms frequently coexist with those of an early intrauterine pregnancy or miscarriage [36].

The primary diagnostic methods are transvaginal ultrasonography and serum levels of beta-human chorionic gonadotropin (β-hCG). However, in cases of pregnancy of unknown location (PUL), where serum β-hCG is detectable but no gestational sac is visible in the uterus or adnexa, interpretation may become more difficult [37]. Although the diagnosis may be supported by serial β-hCG measurements showing an abnormal rise or plateau, high-resolution imaging or diagnostic laparoscopy are frequently required for definitive identification in cases that are unclear [38].

There are serious risks associated with misdiagnosing or delaying the diagnosis of EP, such as internal bleeding, hemodynamic instability, and tubal rupture. Clinicians must therefore keep a high level of suspicion, particularly when dealing with women who exhibit unusual presentations or known risk factors [39].

## 4. Immediate Impact and Management of EP

### 4.1. Types of Management

The size and location of the ectopic mass, serum beta-hCG levels, patient stability, and personal preferences all affect how an EP is managed. The mainstay of medical treatment is methotrexate, an antagonist of folic acid that prevents DNA synthesis and stops trophoblastic tissue growth [40]. Usually, beta-hCG levels are below a certain threshold, usually less than 5000 mIU/mL, and the mass is less than 3.5 cm. Methotrexate is utilized for stable patients with early, unruptured EPs [41]. The drug is injected intramuscularly, and a single-dose or multi-dose regimen may be used. Close monitoring with serial beta-hCG measurements is necessary for patients receiving medical care to ensure the EP resolves and to monitor any possible complications like tubal rupture. Temporary liver enzyme increase, mouth sores, and nausea are common methotrexate side effects [36].

Surgery is required for unstable patients, have ruptured EP, or whose medical therapy has failed or is not appropriate. Salpingectomy and salpingostomy are the two primary surgical alternatives [42]. When the fallopian tube is severely injured or the bleeding is uncontrollable, a salpingectomy—the removal of the afflicted tube—is the preferred procedure. For women who want to preserve their fertility, a more conservative method called a salpingostomy involves making an incision to remove the EP while leaving the fallopian tube intact. However, there is a chance that salpingostomy will result in persisting trophoblastic tissue, which might need additional care [43,44]. Table 2 summarizes the management types of EP and their associated risks.

### 4.2. Emotional and Psychological Consequences Immediately Post-Treatment

The experience of an EP often elicits intense emotional and psychological responses. In addition to shock and anxiety about their current health and future fertility, women may suffer grief and despair akin to those experienced with miscarriage. Anxiety can be greatly increased by the diagnosis’s abruptness and the possibility of potentially fatal repercussions [47].

Many women feel guilt or self-blame in addition to grieving, which is occasionally connected to perceived risk factors like past infections or lifestyle choices. Symptoms of post-traumatic stress disorder could also appear, especially if there was significant bleeding or if emergency surgery was necessary. Women may experience a spectrum of emotional responses, including worry, uncertainty, anger, and grief [48,49].

Compassionate treatment that attends to women’s emotional and physical health is crucial. To assist women in dealing with their loss and anxieties and lower their chance of developing longer-term psychological issues, supportive counseling and mental health referrals can be quite helpful [50].

### 4.3. Short-Term Reproductive Health Effects

The degree of tubal damage and the management strategy employed have a significant impact on the short-term reproductive outcomes of EP and its treatment [51]. Because one fallopian tube is removed during a salpingectomy, fertility may be decreased; however, if the remaining tube is healthy, spontaneous conception may still be feasible. Although there is a minor increase in the likelihood of repeat EP in the preserved tube, salpingostomy saves the afflicted tube, which generally increases the chances of a future pregnancy [46].

Although methotrexate-assisted medical care typically maintains the fallopian tubes’ physical integrity, it is necessary to establish that the ectopic tissue has completely disappeared [52]. Four to six weeks following treatment, the majority of women resume regular menstruation and ovulation. Some temporary problems in menstruation may be caused by methotrexate [53].

To guarantee full resolution, follow-up care involves tracking beta-hCG levels until they reach non-pregnant levels. Additionally, ultrasound can be used to assess fallopian tube healing [54]. To lower the dangers of methotrexate and to give tissue time to heal, women are also frequently recommended to wait at least three months after treatment to become pregnant [55].

Treating EP requires striking a delicate balance between protecting the patient’s safety right away, maintaining fertility when it is feasible, and offering emotional support for the condition’s effects. To maximize physical and psychological results, early and efficient therapy is crucial, as is compassionate post-treatment care [56].

## 5. Impact on Subsequent Fertility

### 5.1. Tubal Factor Infertility

Due to fallopian tube injury or loss, EP frequently impairs fertility. Since the fallopian tubes facilitate fertilization and the transfer of the embryo to the uterus, they are essential for natural conception [57]. Natural conception is less likely when one tube is removed via salpingectomy or is affected by scarring or inflammation [58]. Although many women can conceive with just one good tube, total fertility is decreased. Tubal function can be compromised by previous damage, resulting in tubal factor infertility, even if the tube is retained with conservative surgery, like as salpingostomy [59].

### 5.2. Recurrent EP Risk

Given that recurrence rates are thought to be between 10% and 15%, women who have experienced an EP are more likely to experience one [60]. The degree of tubal damage, the kind of treatment obtained, and underlying illnesses like smoking or pelvic inflammatory disease are some of the factors that raise this risk [31]. The risk of recurrence may be somewhat higher for patients treated with salpingostomy than for those treated with salpingectomy, since the damaged tube is still there. To detect and treat recurrent ectopics as soon as possible, early pregnancies must be closely monitored [33,61,62].

The likelihood of recurrence varies depending on the treatment, with methotrexate falling between 10 and 12% and salpingectomy between 5 and 10% and salpingostomy between 15 and 20%. The psychological toll of “living under recurrence risk” is reflected in the fact that many women report continued anxiety and hypervigilance in subsequent pregnancies, in addition to clinical consequences [63].

### 5.3. Effectiveness of ART

In vitro fertilization (IVF) and other assisted reproductive technologies offer a viable alternative when natural conception is compromised or recurrence risk is elevated. IVF removes the possibility of another tubal pregnancy by fertilizing the egg outside the body and putting the embryo straight into the uterus, avoiding the fallopian tubes [64]. In cases of severe tubal damage or following unsuccessful attempts at natural conception following an EP, IVF is particularly helpful. IVF post-EP success rates are often high, giving women who are still infertile following treatment hope [65].

### 5.4. Statistical Data on Pregnancy and Live Birth Rates

After receiving treatment for an EP, 60–70% of women become pregnant within two years, according to research [36]. Cohorts of 300–2000 women in North America and Europe are the source of reported pregnancy rates of 60–70% and live birth rates of 50–65%. Confidence intervals for live births range from 45 to 70%, indicating significant variability. Therefore, rather than being conclusive, these numbers should be regarded as informative [66].

Based on variables including treatment strategy and tubal health, live birth rates after conception can range from 50% to 65% [67]. Since the fallopian tubes are left intact, women treated medically with methotrexate typically have reproductive outcomes that are comparable to or somewhat better than those who undergo surgery [68]. Salpingostomy patients have a higher chance of recurrent EP, but overall, their fertility prospects are better than those of surgical patients who undergo a salpingectomy [46].

### 5.5. Time to Next Pregnancy and Factors Affecting Delay

Following treatment for an EP, it often takes six to twelve months to conceive [69]. Before attempting to conceive again, women receiving methotrexate are typically recommended to wait at least three months to allow for tissue repair and medication elimination. Access to reproductive care, mental preparedness, the degree of tubal damage, and the existence of additional fertility issues are some of the factors that affect when a woman will become pregnant again [55]. Since emotional stress might postpone attempts to conceive, psychological rehabilitation is also crucial. Women can resume having children sooner if they receive the right support and therapy [70,71].

## 6. Emotional and Psychological Impact on Parenthood Desires

Beyond the initial medical treatment, having an EP can have long-lasting emotional and psychological repercussions. The incident can have a big impact on future reproductive aspirations and decisions for many women and their partners because it signifies both a physical trauma and an emotional loss [72,73].

### 6.1. Post-Traumatic Stress, Anxiety, and Depression After EP

Intense emotional reactions are common in women who have an EP [74]. Similar psychological reactions to those observed following other types of pregnancy loss can be triggered by the abrupt loss of a pregnancy, which is frequently followed by emergency medical procedures or surgery [75]. Instances of flashbacks, nightmares, and hyperarousal are common symptoms of post-traumatic stress disorder (PTSD). Depression and anxiety may also surface, particularly in the weeks and months after the incident [76,77].

It is evident from quantitative research that this burden is not only anecdotal. One month following an EP or miscarriage, 29% of women in a prospective cohort fulfilled the diagnostic criteria for PTSD; at nine months, the frequency had dropped to about 18% [78]. Similarly, within the first three months after a loss, 30–40% of people exhibit clinically significant anxiety or depression symptoms, and up to 20% of these persist for more than a year. Given its abrupt and potentially fatal nature, EP poses a danger to mental health, even though stillbirth is linked to significantly higher and longer-lasting morbidity (30–40% PTSD and depression lasting >12 months) [79].

Women with a history of previous infertility or a long-awaited pregnancy report greater rates of chronic depression, and psychological anguish is most severe following ruptured EP, hemodynamic instability, or an unexpected salpingectomy [2,5]. Beyond diagnostic categories, many women express intense feelings of grief, guilt, or rage, especially when risk factors like smoking or a history of pelvic infections are present and cause self-blame. Pregnancy that is planned or highly desired exacerbates this emotional impact [80].

### 6.2. Impact on Decision-Making Regarding Future Pregnancies

Future pregnancies are frequently viewed differently by women (and couples) after an EP. Concerns about recurrence or future miscarriage can contribute to fear of pursuing another pregnancy. Others, particularly those who were already concerned about fertility, might feel more pressure to become pregnant [81].

The severity of the EP, the type of treatment received, the counsel given by medical professionals, and cultural or personal views on pregnancy loss have an impact on the decision to try for another pregnancy. The ability to enjoy and maintain a healthy pregnancy can be impacted by unresolved trauma or fear, so emotional preparedness is essential [82].

In subsequent pregnancies, some women may seek reassurance through early pregnancy monitoring, while others decide to postpone efforts at conception until they feel emotionally and psychologically mature [83].

Certain cultures offer communal assistance through ritualized mourning, while collectivist societies may stigmatize or suppress reproductive loss. Coping strategies vary depending on the religious interpretation, such as when loss is framed as the will of God. Global relevance is increased when such variety is acknowledged [11].

### 6.3. Coping Mechanisms and Counseling Needs

After an EP, different people cope differently. While some women may struggle in quiet, others may find help in talking to friends, relatives, or participating in online support groups. Grief, trauma, and anxiety can all be processed with the aid of formal psychological counseling or therapy [84].

Following an EP, women who are experiencing extreme emotional distress have demonstrated advantages from cognitive-behavioral therapy (CBT), grief counseling, and trauma-informed treatment. PTSD and persistent depression are two long-term mental health conditions that can be prevented with early psychological care [79,85].

To identify symptoms of mental distress and direct patients to the right support programs, healthcare professionals are essential. Women can cope by receiving written information, scheduling follow-up appointments to talk about emotional well-being, and normalizing the emotional impact [86].

### 6.4. Partner Dynamics and Couple’s Reproductive Planning Post-EP

EPs may have emotional repercussions for partners as well. Especially if the woman’s life was in danger, partners may feel their own grief, concern, or helplessness. Relationship tension can occasionally arise from partners’ different coping mechanisms [87,88].

While some couples have difficulties expressing themselves, conflicting desires for future pregnancies, or changes in their sexual connection due to fear or worry, others become closer through shared sorrow [89,90].

Grief and helplessness are common experiences for partners; men tend to repress their feelings of suffering because of gender standards, while same-sex or non-traditional partners express particular difficulties in acknowledging and disclosing their loss [91]. Relationship consequences range from increased ties via shared resilience to conflict and decreased sexual intimacy. The shame associated with reproductive loss or infertility can exacerbate marital stress and social marginalization in collectivist societies [92]. According to research, men frequently repress their grief because of masculine stereotypes, and same-sex couples may encounter further obstacles to acceptance and assistance. Relationship consequences include increased intimacy through cooperative coping, conflict, and sexual avoidance [93].

Partners can discuss their feelings, loss, and reproductive intentions in an open and honest manner with the support of couple-based counseling. It also offers a forum for talking about worries about future fertility, the dangers of becoming pregnant, and the emotional preparedness for another attempt. Involving both spouses in post-EP care planning can help them make decisions about their reproductive future together and improve their relationship [94].

## 7. Parenthood Pathways Post-EP

After having an EP, being a parent is frequently a difficult and very personal experience. While many individuals or couples still aspire to become pregnant in the future, others deal with fertility issues that persist, psychological suffering, or changes in priorities that affect reproductive choices [95]. The routes to parenthood after EP usually fall into a few general categories: adopting a kid, choosing to live child-free (either voluntarily or involuntarily), using assisted reproductive technologies (ART), and natural pregnancy [96].

### 7.1. Natural Conception Outcomes

Following an EP, many women still have the chance of becoming pregnant spontaneously, especially if their contralateral fallopian tube is still open and functioning [97]. According to studies, assuming there are no serious underlying infertility issues, between 50 and 80 percent of women who try to conceive following an EP will become pregnant again within two years [98]. Maternal age, the degree of tubal damage, the kind of EP treatment (medical versus surgical), and the existence of additional reproductive health issues are all factors that affect a successful spontaneous conception [2,36].

Women who have conservative medication treatment, such as methotrexate, tend to have greater tubal function than those who have surgery, particularly a salpingectomy [99,100]. Comparing salpingectomy to salpingostomy, which preserves the afflicted tube, the former involves a higher risk of recurrent EP, estimated at 10–20%. The risk of recurrence must be weighed against the preservation of fertility in this therapeutic conundrum [46]. In low-income countries, where access to ART is restricted and diagnosis is delayed, post-EP rates are significantly lower. Given that couples frequently blame the previous EP for their repeated infertility, this discrepancy makes trying again more psychologically taxing [101].

Individual differences in time to conception are significant. While some women endure lifelong subfertility, others become pregnant after a few months. Importantly, the psychological effects from an EP might cause worry, unresolved sadness, or fear of recurrence, which can delay efforts at conception even in women with bilateral tubal patency [70,102].

For women who do not conceive within a year of attempting after EP, routine fertility assessment is usually advised, particularly if there are other risk factors for infertility. Referrals to reproductive specialists at an early stage can maximize results and offer pertinent advice [103].

### 7.2. Role of Fertility Treatments

ART is essential for women who become infertile after having an EP, especially if they have a history of multiple EPs or bilateral tubal damage. In these situations, IVF is still the best course of action because it allows embryos to be transferred directly into the uterus, avoiding the fallopian tubes completely [104].

According to several studies, women with a history of EP have IVF success rates that are similar to those of women with other tubal factor infertility reasons. Nonetheless, even with IVF, the chance of recurrent ectopic or heterotopic pregnancy (simultaneous intrauterine and ectopic gestation) is marginally increased, particularly in women who have had considerable past tubal disease [105,106].

In situations when at least one fallopian tube is still open and tubal function is thought to be sufficient, additional fertility treatments like ovulation induction or intrauterine insemination (IUI) may be pursued. To quickly detect any recurring ectopic implantation, however, close observation is recommended in the early stages of pregnancy [107].

Restricted access to ART therapies may be a major obstacle for certain people, especially those with fewer financial resources. Despite improved post-EP IVF success rates, accessibility is still restricted. Although there is some insurance coverage in high-income nations, many people cannot afford the out-of-pocket expenses. ART is frequently inaccessible in LMICs, which exacerbates reproductive disparities [108]. For women who experience infertility following EP, this emphasizes the value of multidisciplinary support, which includes counseling and reproductive planning [109].

### 7.3. Decision for Child-Free Living: Voluntary Versus Involuntary

Individuals or couples may decide to conceive again after an EP. The decision to remain childless may be the consequence of personal choices or involuntary infertility and ineffective reproductive treatments [110,111].

Concerns about the health hazards of becoming pregnant again, a reassessment of life goals, or emotional trauma from the former experience could all be reasons for choosing to live childless following EP. Psychological counseling can facilitate emotional processing and support individuals in accepting their reproductive choices without stigma [110,112].

In contrast, factors such as financial or logistical barriers to reproductive treatments, recurrent miscarriage, and persistent infertility frequently culminate in involuntary childlessness [113]. Many may experience protracted sadness, melancholy, and complex feelings of inadequacy and loss as a result of this. According to studies, women who are unwillingly barren following EP frequently have higher levels of psychological suffering and a lower quality of life than those who become parents through any other way [114,115].

Healthcare professionals must to be aware of the psychological effects of involuntary childlessness and proactively provide options for counseling, peer support groups, and mental health support to help people deal with this result [116].

### 7.4. Adoption Considerations

Adoption provides a different and more fulfilling route to motherhood for some people and couples after they have experienced an EP. The biological bond of a pregnancy cannot be replaced by adoption, but it can satisfy the need to raise and care for a kid [117].

The decision to seek adoption may occur after medical reproductive therapies have been exhausted and frequently comes after a period of mourning for lost fertility. Pre-adoption psychological counseling is frequently advised because there are huge variations in emotional preparedness for adoption [118].

Whether they choose domestic or international adoption, as well as the adoption laws in their country, prospective adoptive parents may have to negotiate complicated legal, financial, and procedural processes. Medical and psychological tests are also frequently required by adoption agencies to determine an applicant’s fitness and preparedness for adoption.

A woman’s medical history or psychiatric assessment may have an impact on her eligibility for adoption. The process is further complicated by cultural differences in acceptability; whilst some societies welcome adoption, others place a greater emphasis on genetic continuity. Developing a new parental identity while grieving for lost fertility is frequently necessary for psychological adjustment [119].

Crucially, those who decide to adopt after EP frequently express happiness with their parenting and positive long-term psychological adjustment. However, the key to assisting patients in exploring this option in a timely and informed manner is early and empathetic counseling by healthcare providers [120].

## 8. Recommendations for Clinical Practice

A woman’s emotional health and future reproductive prospects may be impacted by the long-lasting medical and psychological impacts of EP [121]. Consequently, it is crucial to provide thorough follow-up care that takes into account both psychological and physical factors. Clinicians play a crucial role in helping patients deal with issues related to fertility, future planning for pregnancies, emotional healing, and proper monitoring during subsequent pregnancies [122,123].

### 8.1. Follow-Up Fertility Counseling

All impacted women should be provided with personalized fertility counseling following treatment for an EP. A thorough discussion of how the EP and its treatment (medical vs. surgical) may affect future fertility should be part of the counseling [124,125]. Future factors that may impact tubal function, the likelihood of a spontaneous conception, and the risk of recurrent EP should all be discussed by clinicians. Timely fertility testing is advised for women who wish to become pregnant in the future, particularly if conception does not happen six to twelve months after treatment [126].

Referrals to reproductive medicine specialists for additional examination and potential ART consultation should be made as soon as possible for women with additional risk factors for infertility, such as bilateral tubal injury, advanced maternal age, or a history of pelvic inflammatory illness [127].

### 8.2. Psychological Support Services

EP can have a significant psychological impact; women who experience it are more likely to have feelings of anxiety, despair, bereavement, and post-traumatic stress disorder. As a result, during follow-up visits, clinicians should regularly evaluate patients’ emotional health and, if necessary, refer them to psychological support services. Women can process their experience and lessen long-term psychological discomfort by having access to peer support groups, mental health specialists, and therapy [128,129].

Evidence supports early psychological screening within 4–6 weeks post-EP, with stepped-care models (peer support → CBT → trauma-informed therapy) improving outcomes. In integrated care pathways, mental health professionals work alongside gynecological teams to promote prompt identification and lower long-term morbidity [130].

Early psychological psychotherapy may strengthen coping mechanisms and lessen emotional distress, especially for women who suffer from repeated pregnancy loss or who become infertile after EP. Help-seeking behavior can be encouraged and stigma can be lessened by normalizing conversations about emotional reactions to EP [131].

### 8.3. Preconception Counseling for Future Pregnancy Planning

Prompt pregnancy testing should be recommended for women with a history of EP whenever they miss a menstrual period in the future. To facilitate prompt diagnosis and action, women should be taught the value of obtaining an early medical evaluation in subsequent pregnancies [132].

ESHRE permits earlier tries if hCG is undetectable, but ACOG recommends waiting at least three months after methotrexate before becoming pregnant. Similarly, different locations have different imaging procedures and hCG monitoring intervals. The significance of adapting guidance to local standards is highlighted by pointing out these variations [40,133].

### 8.4. Early Pregnancy Monitoring in Subsequent Conceptions

Women having a history of EP should be closely monitored during the first trimester of pregnancy. To confirm intrauterine gestation and rule out recurrence, this includes early transvaginal ultrasonography and serial serum beta-human chorionic gonadotropin (β-hCG) tests. To localize the pregnancy and reduce the chance of a delayed diagnosis of a recurrent EP, early ultrasonography, usually carried out at around 5–6 weeks of gestation, is essential [134,135].

It is important to be on guard for both ectopic and heterotopic pregnancies in women who become pregnant following reproductive treatments, especially IVF. Early transvaginal ultrasonography helps women in high-resource environments reduce worry, while in LMICs, the lack of early monitoring exacerbates recurrence anxiety. When it comes to conception planning, women’s sense of security is also influenced by insurance coverage of fertility care [136,137]. Coordinated care that takes into account psychological issues as well as medical hazards can be provided via multidisciplinary management that includes obstetricians, reproductive experts, and mental health professionals [138].

## 9. Research Gaps and Future Directions

Although there have been great advancements in the therapy of EP in recent decades, there are still significant gaps in our understanding of and ability to address the long-term psychological and reproductive repercussions that affected persons must deal with. To improve outcomes pertaining to both reproductive and emotional well-being, more research is required to optimize care pathways [139,140,141].

### 9.1. Improved Mental Health Interventions

There is still insufficient evidence-based mental health interventions designed especially for this patient population, despite increased awareness of the psychological effects of EP [87]. The post-miscarriage support models and generalist counseling that are frequently used in clinical practice today may not adequately address the particular emotional difficulties that EP presents, such as fear about future reproductive risks or trauma from emergency surgical procedures [142].

Future studies should concentrate on creating and assessing focused psychological interventions, such as peer support frameworks created especially for women recovering from EP, cognitive-behavioral therapies (CBT), and trauma-informed counseling techniques [143]. Clinical practice would greatly benefit from randomized controlled studies evaluating the impact of early psychological therapies on lowering symptoms of anxiety, sadness, and post-traumatic stress disorder (PTSD). Furthermore, longitudinal research examining women’s long-term mental health outcomes after EP would help determine which women are most vulnerable to persistent psychological suffering [144].

### 9.2. Personalized Risk Assessment for Fertility Outcomes

Nowadays, generalized population-based data are frequently used in fertility counseling following EP, which may not fully represent individual prognoses. A research gap exists in the development of personalized risk assessment models that incorporate various patient-specific factors, including age, the type of EP management, the extent of tubal damage, reproductive history, and underlying gynecological conditions like pelvic inflammatory disease or endometriosis [145,146,147].

More accurate fertility risk classification may be possible with the use of new reproductive medicine technologies like predictive algorithms, blood biomarkers, and sophisticated imaging methods. Clinicians may be better equipped to forecast the likelihood of a spontaneous pregnancy and adjust recommendations for reproductive treatments by incorporating artificial intelligence (AI) and machine learning models [148].

Furthermore, studies on fertility-related quality of life following EP that focus on patient-reported outcome measures (PROMs) may shed light on women’s reproductive priorities and guide collaborative decision-making [149].

### 9.3. Future Directions

Collaboration between researchers, psychologists, fertility specialists, and obstetricians is crucial to filling these gaps. Prioritization should be given to multicenter prospective trials, qualitative research examining patient experiences, and the creation of clinical decision-support tools. Furthermore, incorporating patient perspectives into research design will guarantee that future counseling techniques and interventions are appropriate and relevant for the target population [150,151].

More comprehensive, patient-centered care paths following an EP will eventually be possible with a more detailed understanding of the relationship between psychological health and physical fertility outcomes [152].

## 10. Conclusions

Even after the acute clinical episode, EP is still a major reproductive health event with far-reaching effects. Its effects on psychological health, prospective parental routes, and eventual fertility highlight the necessity of all-encompassing, interdisciplinary therapy. Although a variety of medical, surgical, and personal factors can affect individual reproductive outcomes, many women can still conceive naturally after EP. For many experiencing tubal infertility, assisted reproductive technologies provide useful alternatives; for others, adoption and choosing to live childless are significant, legitimate routes to parenting. However, involuntary childlessness continues to be an unresolved and emotionally taxing situation for some people. The psychological aftereffects of EP are equally significant. In ordinary therapeutic care, feelings of grief, anxiety, and dread of recurrence are widespread and can go unnoticed. As part of follow-up care, this emphasizes the importance of early psychological evaluation and access to mental health support services.

In the future, professional practice should prioritize holistic care models that attend to women’s reproductive, emotional, and physical needs following EP. Preconception planning, early pregnancy monitoring, customized fertility counseling, and access to focused psychological support are all included in this. Existing knowledge gaps must be filled by research, especially in the areas of creating evidence-based mental health treatments and customized fertility risk assessments. The best possible physical and psychological results following an EP can be achieved by providing women with knowledge, emotional support, and individualized treatment paths.

## Figures and Tables

**Table 1:** This table summarizes recognized risk factors for ectopic pregnancy, their proposed mechanisms, and relative risks.

Risk Factor	Mechanism	Relative Risks
Prior EP	Tubal damage, impaired motility [18]	Recurrence risk 10–25% [19]
Pelvic Inflammatory Disease (PID) [20]	Tubal scarring and adhesions [21]	Strong association, especially with Chlamydia
Tubal surgery (e.g., ligation, reconstructive procedures [22]	Altered tubal anatomy	Increased risk varies by procedure
Assisted Reproductive Technologies (ART) [23]	Underlying tubal pathology, embryo transfer technique	Risk higher in IVF-conceived pregnancies
Smoking [24]	Impaired ciliary function and altered tubal motility	Dose-dependent increase in risk
Advanced maternal age (>35 years) [25]	Age-related tubal dysfunction	Moderate risk elevation
Intrauterine device (IUD) failure [26]	Prevention of intrauterine pregnancy but not ectopic implantation	Risk of EP higher if conception occurs with IUD in place
Endometriosis [27]	Pelvic adhesions, tubal dysfunction	Emerging evidence of association
Previous abdominal or pelvic surgery [28]	Adhesion formation	Increases ectopic implantation risk

**Table 2:** Risk factors for ectopic pregnancy, their proposed mechanisms, and associated relative risks.

Management Type	Indications	Procedure	Advantages	Risks
Medical (Methotrexate) [41]	Stable patients with small, unruptured ectopic (<3.5 cm), beta-hCG <5000 mIU/mL	Intramuscular injection of methotrexate to inhibit trophoblastic growth	Non-invasive, preserves fallopian tube Outpatient treatment	Requires careful monitoring Possible side effects (nausea, liver enzyme changes) Risk of treatment failure or rupture
Surgical-Salpingectomy [45]	Hemodynamically unstable, ruptured ectopic, severely damaged tube	Laparoscopic or open removal of the affected fallopian tube	Definitive treatment Eliminates damaged tube	Loss of one fallopian tube, Surgical risks (bleeding, infection, anesthesia)
Surgical- Salpingostomy [46]	Desire for fertility preservation, stable patient with intact tube	Incision on fallopian tube to remove ectopic, preserving tube	Fertility preservation Minimally invasive	Risk of persistent trophoblastic tissue Possible recurrent EP

## Data Availability

No new data were created or analyzed in this study. Data sharing is not applicable to this article.
